# Supplementary material for: Factors Associated with Vaccination Intention against the COVID-19 Pandemic: A Global Population-Based Study
Source: Vaccines (Basel). 2022 Sep 16;10(9):1539. doi: 10.3390/vaccines10091539 (PMC9504242; doi:10.3390/vaccines10091539)
Supplement: Supplementary file 1 [file vaccines-10-01539-s001.zip › vaccines-1876144-supplementary.pdf]

vaccines

MDPI

Supplementary material 1 - Questionnaire

* 1. *Have you ever filled out this questionnaire?*

I have ever filled out it

I have never filled out it

* 2. *Please note that you can stop the survey at any time. This will not entail any penalty, and it will not affect the services (health care services or others) that you receive. By selecting the "agree box", you are agreeing that you are at least 18 years old, that you have read the information about the study, and that you voluntarily agree to take part in it.*

I agree to participate in this study

I do not agree to participate in this study

* 3. *Date*

Date/Time

Part 1: Socio-demography

4. *How old are you?*

5. *What is your sex?*

Male

Female

Other

I prefer not to tell

6. *Which country or region are you currently living in?*

Australia

Thailand

Canada

China

South Korea

Germany

Japan

India

Malaysia

Philippines

Mexico

New Zealand

Ecuador

United Kingdom

United States

Other (please specify)

7. *What is your race?*

American Indian or Alaska Native

Asian
Black or African American
Native Hawaiian or Other Pacific Islander
White
Other (please specify)

8. *How many years of education have you completed (including non-formal education such as part time, night)?*
0-9 years
10-12 years (secondary school completed)
more than 12 years

9. *Where do you live?*
Rural area
Urban area
Rural–urban fringe
Other (please specify)

10. *Who lives in your household besides yourself?*
I live alone
I live with my family members (parents, husband/wife, children, etc)
None of the above

11. *Please indicate your work or study status*
Full-time employed
Part-time employed
Self-employed
Not employed but not student
Student
Retired
Other (please specify)

12. *Please indicate your health insurance coverage.*
None
Partial coverage
Full coverage (public)
Full coverage (private)

13. *Please indicate whether you receive welfare benefits.*
Yes
No

Part 2: Medical conditions

14. *Have you ever been vaccinated against influenza?*

Yes

No

15. *Please indicate which of the following conditions apply to you (Yes/no)*

Cardiovascular disease (e.g. coronary heart disease, heart failure, cardiomyopathy)

Hypertension

Type 2 Diabetes

Immunodeficiency, or taking medication that suppresses the immune system (e.g. corticosteroid)

Chronic disease of the respiratory system (e.g. asthma, chronic bronchitis)

Chronic liver disease

Chronic kidney disease

Cancer during past 5 years

Sickle cell disease

16. *Have you ever been diagnosed by a doctor or therapist with one or more of the following? (Yes/ No)*

Depression

Mania/Bipolar disorder

Psychotic disorders (including schizophrenia)

Anxiety disorder

Posttraumatic stress disorder

Eating disorder

Compulsive disorders (OCD)

Substance abuse or Addiction disorder

Attention disorder (ADD or ADHD)

Somatoform disorder

Personality disorder

Autism Spectrum

Disorder (including Asperger's Syndrome)

Cognitive disorder/dementia

Part 3: Perception related to vaccination intetnion

17. *If a COVID-19 vaccine is made available in my country, my decision of whether or not to get vaccinated would depend on: (Strongly disagree, disagree, neutral, agree, strongly agree)*

Country in which the vaccine is produced.

Recommendation from my family doctor.

Recommendation of the Ministry of Health.

Whether the vaccine has been in use for 2 years or more.

Whether the vaccine with no serious side effects.

Whether the vaccine is used in other countries.

My risk of getting infected with COVID-19.

How easy it is to get the vaccine (e.g. available out-of-hours or in pharmacies).

Whether the vaccine is free of charge.

Whether restrictions on movement and gathering in groups would be lifted if most people got the vaccine.

Supplementary Table S1. Number of responses by country.

Country	N
Philippines	893
China	483
United States	285
Ecuador	220
Thailand	192
Mexico	177
Australia	57
Canada	45
Kingdom of Saudi Arabia	15
Japan	9
South Korea	9
Malaysia	9
New Zealand	4
France	3
United Kingdom	2
Singapore	2
Switzerland	1
India	1
Colombia	1
Nigeria	1
Germany	1
Oman	1
Italy	1
Indonesia	1
Taiwan	1
Pakistan	1
United Arab Emirates	1
Peru	1
Nepal	1
Central America	1
Iraq	1
Russia	1
Missing	38
**Total**	**2459**

Supplementary Table S1b. Organization of the national health system and style of local communication by country.

Country	Style of Local Communication	Organization of the National Health System	Healthcare Coverage
Philippines	Filipino, English	Department of Health - Republic of the Philippines[1]	All Filipinos are members of the National Health Insurance Program. During COVID-19 pandemic, fees for laboratory testing, community isolation, and hospitalization are covered.[6]
China	Chinese	National Health Commission of the PRC[2]	After the healthcare reform, China has achieved near-universal health coverage (more than 95% of the population). [7]
United States	English and Spanish	U.S. Department of Health & Human Services[3]	The US does not have a single nationwide system of health insurance. Health insurance can be purchased in the private market or is provided by the government to certain groups.[8]
Ecuador	Spanish	Ministerio de Salud Pública *[Ministry of Health]*[4]	Ecuador has carried out a healthcare reform which reduced unmet healthcare needs to 18% in 2014 from 27% in 2006. All MoH health services became progressively free for all citizens.[9]
Thailand	Thai	The Ministry of Public Health[5]	Thailand has had universal health coverage since 2002, and policy was made in 2020 to fully fund a comprehensive benefit package for COVID-19 which includes laboratory tests, clinical service, vaccination costs, etc.[10]

1. https://doh.gov.ph/
2. http://en.nhc.gov.cn/
3. https://www.hhs.gov/
4. https://www.salud.gob.ec
5. http://www.moph.go.th
6. Haw NJL, Uy J, Sy KTL, Abrigo MRM. Epidemiological profile and transmission dynamics of COVID-19 in the Philippines. Epidemiology and Infection. Cambridge University Press; 2020;148:e204.
7. Yue X, Li Y, Wu J, Guo JJ. Current Development and Practice of Pharmacoeconomic Evaluation Guidelines for Universal Health Coverage in China. Value Health Reg Issues. 2021 May;24:1-5. doi: 10.1016/j.vhri.2020.07.580. Epub 2020 Dec 19. PMID: 33349598.
8. Ridic G, Gleason S, Ridic O. Comparisons of health care systems in the United States, Germany and Canada. Mater Sociomed. 2012;24(2):112-20. doi: 10.5455/msm.2012.24.112-120. PMID: 23678317; PMCID: PMC3633404.
9. Quizhpe, E., Teran, E., Pulkki-Brännström, AM. et al. Social inequalities in healthcare utilization during Ecuadorian healthcare reform (2007–2017): a before-and-after cross-sectional study. BMC Public Health 22, 499 (2022). https://doi.org/10.1186/s12889-022-12884-9

10. Tangcharoensathien V, Sachdev S, Viriyathorn S, et al. Universal access to comprehensive COVID-19 services for everyone in Thailand. BMJ Global Health 2022;7:e009281.

Supplementary Table S2. Socio-demographic factors associated with COVID-19 vaccination intention (sensitivity analysis with regions).

Variables	Multiple Logistic Regression Analysis aOR (95%CI)	*P**
**Regions**		
Americas	1 (ref)	
Asia Pacific	0.94 (0.57 - 1.54)	0.794
European	0.87 (0.10 - 8.00)	0.902
Middle East	1.08 (0.13 - 9.01)	0.941
**Age years**	1.04 (1.02 - 1.05)	<0.001*
**Sex**		
Male (ref)	1 (ref)	
Female	1.31 (1.01 - 1.71)	0.044
**Race**		
Asian (ref)	1 (ref)	
White	0.90 (0.51 - 1.61)	0.728
Black	1.05 (0.57 - 1.94)	0.882
American Indian or Alaska Native	1.63 (0.71 - 3.74)	0.246
Others	1.48 (0.86 - 2.55)	0.155
**Years of Education**		
0-9 years (ref)	1 (ref)	
10-12 years	1.63 (0.91 - 2.92)	0.102
> 12 years	1.89 (1.11 - 3.24)	0.020*
**Residence**		
Urban area (ref)	1 (ref)	
Rural area	1.18 (0.88 - 1.60)	0.274
Rural–urban fringe	1.63 (1.00 - 2.65)	0.048*
**Living status**		
live alone (ref)	1 (ref)	
live with family	1.07 (0.72 - 1.58)	0.744
live with other people	0.82 (0.47 - 1.43)	0.486
**Work/study status**		
Full-time (ref)	1 (ref)	
Part-time/self employed	0.62 (0.42 - 0.93)	0.021*
Students	1.67 (1.18 - 2.37)	0.004*
Others	0.44 (0.27 - 0.72)	0.001*
**Health insurance coverage**		
None	1 (ref)	
Partial coverage	0.99 (0.70 - 1.40)	0.932
Full coverage (public)	1.82 (1.28 - 2.59)	0.001*
Full coverage (private)	2.01 (1.30 - 3.09)	0.002*
**Welfare benefits**		
Yes	0.54 (0.41 - 1.71)	<0.001*
No	1 (ref)	
**Mental illnesses**		
Yes	2.27 (1.75 - 2.96)	<0.001*
No (ref)	1 (ref)	

*Significant at $p < 0.05$
